# A brain metastasis liquid biopsy: Where are we now?

**DOI:** 10.1093/noajnl/vdae066

**Published:** 2024-05-02

**Authors:** Stephen David Robinson, James de Boisanger, Frances M G Pearl, Giles Critchley, Nicola Rosenfelder, Georgios Giamas

**Affiliations:** Sussex Cancer Centre, University Hospitals Sussex NHS Foundation Trust, Brighton, UK; Department of Biochemistry and Biomedicine, School of Life Sciences, University of Sussex, Falmer, Brighton, UK; Neuro-Oncology Unit, The Royal Marsden Hospital NHS Foundation Trust, London, UK; Department of Biochemistry and Biomedicine, School of Life Sciences, University of Sussex, Falmer, Brighton, UK; Department of Neurosurgery, University Hospitals Sussex NHS Foundation Trust, Brighton, UK; Section of Neurosurgery, Department of Surgical Sciences, Dunedin School of Medicine, University of Otago, Dunedin, New Zealand; Neuro-Oncology Unit, The Royal Marsden Hospital NHS Foundation Trust, London, UK; Department of Biochemistry and Biomedicine, School of Life Sciences, University of Sussex, Falmer, Brighton, UK

**Keywords:** brain metastases, circulating tumor cells, circulating tumor DNA, extracellular vesicles, liquid biopsy

## Abstract

Brain metastases remain a challenging and feared complication for patients with cancer and research in this area has lagged behind research into metastases to other organs. Due to their location and the risks associated with neurosurgical biopsies, the biology underpinning brain metastases response to treatment and evolution over time remains poorly understood. Liquid biopsies are proposed to overcome many of the limitations present with tissue biopsies, providing a better representation of tumor heterogeneity, facilitating repeated sampling, and providing a noninvasive assessment of tumor biology. Several different liquid biopsy approaches have been investigated including circulating tumor cells, circulating tumor DNA, extracellular vesicles, and tumor-educated platelets; however, these have generally been less effective in assessing brain metastases compared to metastases to other organs requiring improved techniques to investigate these approaches, studies combining different liquid biopsy approaches and/or novel liquid biopsy approaches. Through this review, we highlight the current state of the art and define key unanswered questions related to brain metastases liquid biopsies.

Brain metastases are one of the most common and devastating complications for patients with cancer. Most frequently occurring in patients with non-small cell lung cancer (NSCLC), melanoma and human epidermal receptor 2 positive (HER2) and invasive lobular carcinoma subtypes of breast cancer,^1,2^ they can develop in up to 26% of all patients who die from cancer.^3^ Additionally, with improvements in survival and systemic disease control over the last few decades,^4–6^ alongside more sensitive imaging techniques allowing the identification of even smaller brain metastases,^7^ there has been a significant increase in the identification of brain metastases,^1^ including asymptomatic brain metastases. Brain metastases therefore remain an increasingly significant health burden for patients and the health service.

While survival has dramatically improved for patients with metastatic disease to other organs,^4–6^ the survival of patients with brain metastases has improved yet remains poor,^1,2^ and survival is worse than metastatic disease to other organs. Typically used as an exclusion criterion from clinical trials, brain metastases-related research is extremely limited with significantly fewer research studies than would be expected based on their incidence.^2,8^ However, clinical practice has progressed with the development of stereotactic radiosurgery (SRS), and SRS is now the dominant treatment modality for smaller or multiple brain metastases.^2,9^

## The Need for a Better Biomarker

The decision regarding the most effective choice of treatment for an individual patient with brain metastases relies heavily on the ability to predict response and to tailor treatment based on prognosis. For example, the pivotal QUARTZ trial^10^ demonstrated that the benefit of whole-brain radiotherapy for non-small cell lung cancer brain metastases was dependent on the graded prognostic assessment score. There have been several iterations of validated prognostic tools for brain metastases over recent years, with improvements in treatments for specific subtypes of patients with brain metastases (eg anti-HER2 targeted therapies for HER2 positive breast cancer) requiring the development of the disease-specific graded prognostic assessment tools.^2,8,11–13^ However, these tools are known to overestimate survival compared to population-based registries,^2^ are based on pretreatment factors alone without incorporating a response to treatment and have only recently incorporated basic molecular features.^12,13^ The development of a more nuanced and personalized prognostic tool would therefore provide significant benefits to patients with brain metastases and clinicians.

Additionally, while SRS is generally well tolerated, neurocognitive complications can occur with a reported incidence of between 5% and 35%.^2,14^ This is especially important in those patients with a better prognosis, as the development of neurocognitive complications can have a significant impact on quality of life. The early identification of neurocognitive complications post-SRS treatment as well as an improved ability to predict who might develop neurocognitive effects is therefore a key research priority.^15,16^

Despite the increasing identification of asymptomatic brain metastases,^7^ and the knowledge that treatment of smaller brain metastases has better outcomes,^17^ there are currently no validated screening programmes for patients at higher risk of developing brain metastases.^8,18,19^ Additionally, while the diagnosis can be inferred from imaging findings, confirmation and biological subtyping rely on an invasive neurosurgical brain biopsy. However, currently, the indication for neurosurgical biopsy is limited to patients with brain metastases and an unknown or undetectable primary tumor. Additionally, brain metastasis resection is indicated in patients with a solitary brain metastasis, or occasionally a limited number of brain metastases, when the primary tumor is known to relieve mass effect which facilitates further treatments. Therefore, most patients with brain metastases rely on the inference from imaging findings and prior histology to guide treatment, despite the known discordance in genetic changes between the primary tumor and brain metastases.^20^

**Figure 1.**
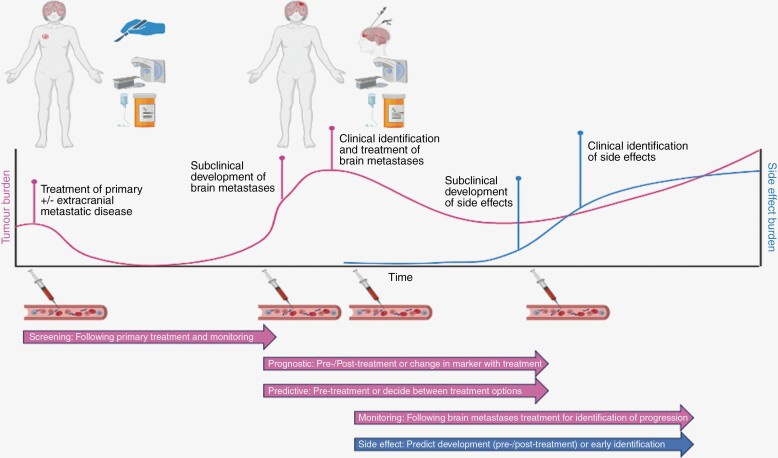
Potential uses of a brain metastases liquid biopsy. Created with BioRender.com.

Following diagnosis and treatment, patients enter magnetic resonance imaging (MRI) surveillance which is an expensive, time-consuming, and resource-intensive process and frequently provides inconclusive findings regarding the differential of tumor recurrence or treatment effects (radionecrosis).^21^ Therefore, there is a clear need for a cheaper and simpler means of diagnosing and monitoring brain metastases at multiple points in the patient pathway, which can also provide information about the underlying tumor biology,^22,23^ as demonstrated in Figure 1.

In this review we discuss the different factors that need to be considered when investigating liquid biopsies for brain metastases, highlight the current evidence covering circulating tumor cells (CTCs) circulating nucleic acids, extracellular vesicles (EVs), and tumor-educated platelets (TEPs), and suggest key unanswered questions for the field of liquid biopsy for brain metastases to develop.

## Liquid Biopsy Options

Liquid biopsies, the assessment of tumor biomarkers in a variety of biofluids including blood components, tears, urine, and cerebrospinal fluid^24,25^ are suggested to provide this information without the need for an invasive, and expensive, tissue biopsy.^26^ There are several different liquid biopsy techniques, which interrogate different analytes, in various stages of development as possible tumor-related biomarkers. The most well-studied analytes, CTCs and either total cell-free DNA (cfDNA) or circulating tumor DNA (ctDNA), have validated and FDA-approved uses in different settings in oncology^27,28^ although their use in neuro-oncology settings has lagged behind their use with other solid tumors. Additionally, a variety of alternate liquid biopsy analytes have been proposed, including EVs^29^ and TEPs,^30^ with their advantages and disadvantages summarized in Table 1.

**Table 1.** Comparison of the Different Analytes Available for Liquid Biopsy Analysis

Analyte	Advantages	Disadvantages
Circulating tumor cells (CTCs)	Significant experience investigating CTCs in other settings. CTC cargo can be interrogated to provide biological information.	Least stable requiring almost immediate processing. Reduced levels due to difficulty transversing the blood–brain barrier.
Circulating nucleic acids (cfDNA, ctDNA, and RNA)	Significant experience investigating circulating nucleic acids in other settings. Can provide evidence for specific mutations related to treatment options or treatment resistance.	Difficult to identify and isolate brain metastasis-derived circulating nucleic acids from systemic metastases. Related to dead/dying tumor cells rather than active cells.
Extracellular vesicles (EVs)	Improved stability and concentration in peripheral circulation. Multi-omic analyses can be performed to identify EV subtypes specific to brain metastases. Provides biological insight into active tumor intercellular signaling.	More complicated separation steps are required (although improving). Require characterization steps prior to biomarker investigation.
Tumor-educated platelets (TEPs)	Highly present in the peripheral circulation.	Currently limited to highly focused laboratories.

An additional variable to consider is the starting biofluid that will be analyzed for the proposed liquid biopsy biomarker, with different biofluids having different advantages and disadvantages as shown in Table 2. The most consistently investigated biofluids across the spectrum of solid tumor oncology are plasma and serum, with the choice dependent on the specific analyte investigated. However, in addition to the choice of blood component, both the specific blood collection tube used as well as the preanalytical processing steps, can have significant effects on the yield and quality of the desired biomarker.^31,32^ This requires careful and accurate documentation of the preanalytical processes in any biomarker identification study and is a significant challenge in attempting cross-study comparisons.

**Table 2.** Comparison of Different Starting Biofluids for Liquid Biopsy Analysis

Biofluid	Advantages	Disadvantages
Blood (plasma and serum)	Well-established source of liquid biopsy biomarkers. Repeated collection is simple and acceptable to patients.	Complexity of biofluid. Multiple different preprocessing decisions.
Cerebrospinal fluid	Direct contact with brain/brain metastases. Much less complex biofluid than blood components.	Technically challenging to collect. Significantly more invasive collection procedure.
Urine	Suitable for self-collection of biofluid. Repeated collection is extremely simple.	May be less relevant for brain metastases. Difficult to normalize collection volumes.
Tears, saliva, etc.	Suitable for self-collection of biofluid. Repeated collection is extremely simple.	Limited collection volumes.

However, blood components are extremely complex biofluids^33^ with a significant degree of noise that requires additional processing steps prior to any biomarker identification studies. For brain tumors, including brain metastases, due to their location, one available option is to analyze cerebrospinal fluid (CSF) for potential biomarkers. CSF is a much less complex biofluid than serum or plasma and as it has close or direct contact with the brain and the tumor such would be expected to have a greater biomarker signal with less noise.^34^ However, the collection of CSF is an invasive procedure, albeit less invasive than neurosurgery, which limits its accessibility and the repeatability of collection. Additionally, CSF is not a homogenous fluid and the site of CSF collection whether directly at neurosurgery, via an Ommaya reservoir, or from different vertebral level lumbar punctures has been shown to alter the CSF contents.^35^ As such a standardized CSF collection and processing is required for improved reliability and to reduce inter-study variability.^36^

Alternatively, other body fluids can be chosen as the starting material for analysis including urine, tears, and saliva. All these biofluids have the advantage that collection is extremely simple and noninvasive, which means that they can be performed at home by the patient and so this facilitates repeated self-collection. However, both saliva and tear fluid can only be collected in very small volumes which has a significant impact on subsequent biomarker yield, whereas there is no single concentration of urine which makes inter-patient comparison difficult. As such there is no single “best” biofluid with trade-offs necessary in the choice of starting biofluid for biomarker investigations and an understanding of the relevant factors necessary for appropriate mitigation steps in preanalytical processing.

The time point when the sample is collected will also impact the potential use of any identified liquid biopsy. For example, post-treatment levels or changes in biomarker levels with treatment might provide greater depth to a prognostic biomarker than a pretreatment level alone. This makes biomarker identification challenging, as traditionally samples have been collected at a single time point which may not be optimal for the required usage. Optimally, patients will undergo longitudinal sampling at several time points in their diagnostic process, treatment, and follow-up to allow for serial assessment of the liquid biopsy analyte. Additionally, the collection and storage of different biofluids with consistent preanalytical processes at each time point, for example collecting both serum and plasma, will provide opportunities to compare different liquid biopsy techniques from the same cohort of patients.

## Circulating Tumor Cells

CTCs were first described in the literature over 150 years ago after an autopsy identified tumor cells in the blood of a man with metastatic cancer.^37^ Today, liquid biopsies that examine CTCs are being increasingly used across many tumor subtypes.^38,39^ CTCs represent an intermediate phase in the development of tumor metastases, being able to migrate from the tissue of origin, intravasate and circulate in the bloodstream.^40^ Depending on the stage of cancer, they may also be able to extravasate and form distant metastatic colonies, from which, in turn, CTCs can return to the bloodstream.^38^ In this way CTCs have the potential to inform us about the primary tumor, metastasis development, and the sites of metastatic disease themselves, including BMs.

CTCs represent only a very small proportion of circulating cells, and successful detection assays rely on isolating and collecting them from among the significantly more numerous surrounding leukocytes.^40,41^ CellSearch, using ferromagnetic EpCAM antibodies, remains the only FDA-approved methodology for the enumeration of CTCs in blood, having been initially approved in 2004.^42^ However, other detection technologies are now in use and development, including microfluidics, which takes advantage of small differences in size, geometry, and density to separate cells by ultra-precise manipulation of fluid flow.^40,43^

To date, CTCs have shown promise for a variety of clinical uses in non-BM settings.^38^ In early stage cancers, CTC enumeration has been used for prognostication,^44–46^ risk stratification for adjuvant treatments,^47^ and post-treatment surveillance.^48^ In advanced metastatic cancer, CTCs have also been shown to be prognostic, both at diagnosis^49,50^ and in response to treatment.^51^ However, the role of CTC detection in assisting treatment decisions has been largely disappointing when tested in clinical trials.^52,53^ As well as the detection and enumeration discussed above, CTC molecular characteristics can also be assessed, offering a window into tumor biology and clonality. A landmark clinical trial published earlier this year demonstrated a survival advantage for patients with triple-negative metastatic breast cancer treated with anti-HER2 therapy on the basis of HER2+ve CTCs.^54^

However, in patients with brain tumors, the blood–brain and blood–CSF barriers pose significant challenges for CTC-based liquid biopsies, with tight junctions limiting transcytosis into the liquid media.^22,39,55^ However, brain-tumor-derived cells are detectable and quantifiable in CSF through standard cytological examination, with a long-established role in the diagnosis of leptomeningeal disease.^22,55–57^ Brain tumor-derived-CTCs are more readily detectable in CSF than peripheral circulation.^22,55^ In one particularly clear comparison, Le Rhun et al.^57^ demonstrated that using the CellSearch system to analyze 5 mL of CSF collected at the time of intrathecal treatment of melanoma leptomeningeal metastases identified between 5 and 1090 CTC/5 mL compared to no CTC identified from 7.5 mL of blood, although this was assessed in only 2 patients. The same group found similar findings in both breast cancer^58^ (*n* = 8) and non-small cell lung cancer patients^59^ (*n* = 18) with leptomeningeal disease. Unfortunately, as highlighted above, lumbar punctures are uncomfortable for patients and more resource-intensive for hospitals, whereas peripheral blood sample is minimally invasive and convenient.

Through advancements in sample processing and analysis techniques, a clinically validated blood-based detection assay has now been developed for glial malignancies.^60^ Utilizing a proprietary CTC enrichment medium which permits the survival of malignant cells but causes nonmalignant cells to undergo apoptosis, O’Neill et al. investigated the expression of GFAP and OLIG2 to identify gliomas, and in a subset of samples also cytokeratin expression to identify BM patients, on the isolated CTCs from several prospective sample collection studies. While their focus was on identifying glioma patients, which they were able to do with a cumulative sensitivity/specificity/accuracy of >99%, they were able to identify and differentiate 24 BM patients from 40 glial malignancy patients, 22 nonmalignant brain tumor patients, and 500 healthy volunteers with 100% specificity and 100% sensitivity. However, it is unknown whether this test would differentiate patients with BM from patients without BM from the same underlying tumor type. These promising early results raise the prospect that, following further development and testing, such assays may be used for patients with BMs.

A particular challenge for the development of BM-specific CTC assays is our incomplete understanding of the brain metastatic process and the features which uniquely distinguish BM CTCs from other metastatic CTCs from the same primary.^39^ This is a particular challenge for BMs as, due to impaired migration through the BBB, the proportion of circulating CTCs which are BM-derived is likely to be much lower than that from extracranial systemic metastases. Additionally, many of the most widespread CTC assays use EpCAM antibodies to separate cells of epithelial (ie CTCs) and hematopoietic (ie leukocytes) origin,^38^ however, EpCAM-negative populations of tumor cells have been shown to have high brain metastatic potential^23,61^ and so the isolation of EpCAM-negative CTCs therefore requires alternate approaches.

Zhang et al.^61^ identified EGFR overexpressing/heparanase positive/aldehyde dehydrogenase 1 (ALDH1) positive circulating peripheral mononuclear cells in 8 breast cancer patients without EpCAM-positive CTCs which they defined as potentially metastatic CTCs. Following FACS selection of ALDH1 positive/CD45 negative (a common hematopoietic cell marker) CTCs, they were able to separate and propagate primary CTC cultures from 3 of the 8 patients. Through assessment of a targeted panel of known BM-associated genes, they proposed a BM signature (CTCs positive for heparanase, Notch1, EGFR, and HER-2) which they found had a propensity for BM development in a murine model. Boral et al.^23^ took a different approach to identifying EpCAM-negative CTCs and performed multi-parametric flow cytometry using the DEPArray followed by RNA sequencing to define a transcriptomic signature of EpCAM-negative CTCs from 10 patients with breast cancer patients. A subsequent analysis of 5 patients with detectable BM compared to the 5 patients without BM demonstrated gene expression differences which clustered the patients based on the presence of BM and derived a 126-gene signature. Interestingly, a greater proportion of CTCs from BM-positive patients demonstrated positive Notch1 staining, again highlighting Notch signaling as a mechanistic pathway in the development of BM, at least for patients with breast cancer.

Furthermore, the utility of simple CTC enumeration for predicting the presence or development of BMs seems limited. For example, Naito et al.^62^ demonstrated there was no association between the number of CTCs and the presence of BMs in 51 patients with small-cell lung cancer. However, they enrolled a heterogenous cohort of patients and included only 7 patients with baseline BM and were only able to identify CTCs in 68.6% of patients using the CellSearch system, both of which limit the statistical power to make definitive results. On the contrary, in a preplanned secondary analysis of 44 patients in the LANDSCAPE trial which investigated lapatinib and capecitabine as first-line treatment of HER-2 positive metastatic breast cancer in patients with BM who had not received whole brain radiotherapy, Pierga et al.^63^ also used the CellSearch system to demonstrate that early clearance of CTCs after 1 cycle of treatment could predict intracranial response and overall survival. However, a decrease in CTC count after 1 cycle of treatment was identified in both responders and nonresponders and while the change in CTC count was not associated with extra-cranial response, given only 14.6% of patients had BM-only disease there is a reasonable possibility that change in CTC count relates more to systemic disease control than not specifically related to BM biology. Therefore, significant further research is required for the development of a BM-specific CTC detection assay to allow the potential of CTC liquid biopsies to be realized for patients with BMs.

## Circulating Nucleic Acids

It is with circulating nucleic acids, namely DNA, that the term “liquid biopsy” has been most closely associated.^55^ Circulating cfDNA was first identified in 1948 in France by Mandel and Matais.^64^ However it wasn’t until the 1970s and 80s when cfDNA was found in the plasma of cancer patients,^65,66^ that the field underwent a step change and subsequently cfDNA was first used to detect oncogenic mutations in 1994.^67,68^ Specifically, ctDNA refers to the tumor-derived subset of cfDNA,^69^ which is shed from cancer cells during necrosis or apoptosis,^70,71^ although the precise release mechanisms are still incompletely understood.^72^ The genetic makeup of the ctDNA reflects the cell of origin, and the half-life in circulation is under 3 h^73^ meaning the ctDNA offers a “real-time” window into whole-body disease burden,^70^ although, as it represents dead or dying cells, this may not be representative of the active disease process at that time.

Challengingly for its use as a liquid biopsy, ctDNA is often present in very small quantities and typically makes up <1% of total cfDNA.^74^ This has historically been a significant limitation, as older detection methods such as Sanger sequencing lacked sensitivity to reliably detect ctDNA. However, this has been largely overcome with newer techniques such as digital polymerase chain reaction (dPCR), including its most sensitive variant, BEAMing (Beads, Emulsion, Amplification, and Magnetics)^74,75^; and next-generation sequencing (NGS) with sensitivity in excess of 95%.^69,70,75^ As with CTCs, the clinical utility of assessing ctDNA as a liquid biopsy is expanding rapidly in the non-BM setting,^76,77^ including early detection,^78^ presence of micro-metastatic disease,^79^ treatment selection in advanced disease,^80,81^ and monitoring of clonal evolution.^82^

In BMs, there is the added challenge of the BBB impairing CNS-derived ctCNA from accessing the peripheral circulation,^76,83^ with evidence suggesting that CSF samples provide more reliable information compared to plasma.^34,84^ Clinical application of ctDNA liquid biopsy in patients with BMs is, therefore, more limited than for other conditions with a similar incidence, although several studies have started to explore emerging roles. Lee et al.^85^ monitored ctDNA in 72 metastatic melanoma patients with BMs receiving immunotherapy with PD1 inhibitor therapy. Patients had ctDNA measured at baseline and longitudinally over the first 12 weeks of treatment, 13 patients had intracranial disease only and 59 had both intra- and extra-cranial disease. Undetectable ctDNA at baseline and on treatment was significantly associated with improved survival (HR 0.51, *P* = .03 and HR 0.32, *P* ≤ .01, respectively). However, although ctDNA was detectable in 64% of those with extracranial disease, it was undetectable in all cases of intracranial-only metastases. Therefore, despite plasma ctDNA being prognostic, it was not able to detect or monitor intracranial disease activity. Liang et al.^86^ echoed this in their NGS study of peripherally collected ctDNA in patients with gliomas and BMs. They analyzed ctDNA mutations in 28 patients, 21 with glioma and 7 with BMs, and found distinct subsets of mutations in both groups. However, although ctDNA was detectable in 10 (47.6%) glioma patients, it was only detectable in 2 (28.3%) patients with BMs.

The ability to detect BM-specific ctDNA is important as there is evidence of discordance between the molecular alterations present in brain metastases and in the primary tumor.^20^ As mentioned above, CSF sampling may be a more reliable way of obtaining BM-derived ctDNA.^87^ Wu et al.^88^ performed parallel genomic analyses in matched BM and primary tumor DNA, plasma ctDNA and CSF ctDNA in 20 patients. They found that CSF analysis was able to detect all BM mutations in 83.33% of patients compared to 27.78% for plasma ctDNA. Additionally, Li et al.^89^ collected matched CSF and plasma samples at baseline, 8 weeks after treatment, and at progression in 92 patients with newly diagnosed metastatic NSCLC. NGS demonstrated that CSF-derived ctDNA better predicted intracranial response compared to plasma (concordance of 76% with MRI brain imaging for CSF ctCDNA, compared to 50% for plasma ctDNA). Furthermore, in cases with opposing intra- and extracranial responses, the changes in CSF and plasma ctDNA followed the same trends as the intra- and extra-cranial responses respectively. This indicates that CSF ctDNA may better represent BMs, whereas plasma ctDNA predominantly reflects extracranial disease. Nevertheless, plasma-based assays, with clinically meaningful sensitivity and specificity, have been tested in primary brain tumors,^90^ raising the hope for BM assays in the future.

CtDNA analysis is not limited to the coding portion of DNA, and epigenetic alterations can also be detected, which has resulted in a liquid biopsy based on cfDNA methylation signatures being developed as a multi-cancer early detection test.^78^ For patients at risk of BMs, methylation patterns could aid early BM detection and help to identify the BM primary tumor.^91,92^ Barciszewska^93^ collected brain tumor tissue in 139 patients with matched peripheral blood samples from 45 patients and analyzed 5-methylcytosine (5-mC) content in the DNA. They found that differences in 5-mC content were significantly associated with a primary tumor of origin (*P* = .0001), and negatively correlated with tumor grade (*P* = .017). Importantly, 5-mC content in matched peripheral samples and brain tumor tissue was not different (*P* = .58) and were moderately strongly correlated (*r* = 0.69).

Nucleosome positioning, so-called fragmentomics,^94^ is another molecular characteristic which can be analyzed in ctDNA, with promising results in distinguishing cell and tissue of origin. Snyder et al.^95^ examined nucleosome footprints in cfDNA from 5 patients with metastatic cancer (lung, liver, breast, and colorectal), finding that some of the most commonly present fragments represented cancer cell lines, in contrast to 3 healthy controls where almost all of the top 20 were hematopoietic lineages. Notably, the fragments correctly indicated cancer of origin in 3 out of 5 (60%) patients with cancer. Such techniques may find utility in the brain metastatic setting in the future.

Liquid biopsies based on non-DNA nucleic acid targets also exist. However, RNA is unstable in the blood which limits its current role as a target for liquid biopsy.^72^ Nevertheless, RNA transcripts, especially noncoding RNA, play a key role in tumorigenesis by regulating the gene expression of pathways such as the cell cycle and apoptosis.^96^ Some types of noncoding RNA, notably microRNA (miRNA) and long noncoding RNA are stable to RNase degradation and detectable in blood, and there is increasing research into their use as a liquid biopsy biomarker.^72,96^ Xu et al.^97^ identified 2 miRNAs associated with leptomeningeal disease from matched serum and CSF samples in 4 patients with leptomeningeal disease secondary to NSCLC and subsequently validated these findings in a further cohort of 30 patients (7 with leptomeningeal disease and 23 without leptomeningeal disease). Importantly, the overall expression levels of miRNA were higher in serum than in CSF, leading the authors to conclude that their serum assay could replace CSF sampling to predict leptomeningeal disease in NSCLC. Additionally, a novel form of RNA called circular RNA is emerging as an additional mechanism regulating the development of brain metastases, including circBCBM1,^98^ and there are ongoing investigations into their potential as a liquid biopsy.^99^

## Extracellular Vesicles

One more novel source of blood-based biomarkers, EVs, has several distinct theoretical advantages over the other analytes previously discussed,^100^ including circulating nucleic acids and CTCs, due to the stability and relative abundance of EVs in the peripheral circulation.^29^ EVs are small, lipid bilayer-enclosed particles containing bioactive materials involving proteins, lipids, nucleic acids (DNA, micro-RNA and long noncoding RNA (lncRNA)), and metabolites.^101^ Among their many proposed functions to date, EVs have the potential to contribute to intercellular signaling by transferring their bioactive cargo.^102^ Cancer cell-derived EVs are associated with many cancer hallmarks,^103^ implicated in establishing and developing the tumor microenvironment (TME) through facilitating bidirectional oncogenic signaling with surrounding nonmalignant cells,^104^ while they have also been identified as a mechanism of treatment resistance.^105^ EVs have also been implicated in several aspects of the metastatic cascade^106^ including acting as a key determinant of the tropism of metastases to different organs.^107,108^

EVs require active secretion from living cells, while their lipid bilayer membrane protects their cargo from degradation.^109^ These features, alongside their short half-life of only a few hours,^110^ their ability to reflect their cell of origin,^111^ and their presence in the peripheral circulation,^112^ raise the possibility that they could be used as dynamic liquid biopsy biomarkers for various tumor types.^113,114^ Within the last few years, there have been several clinical or translational studies focusing on EVs as either a liquid biopsy biomarker or to understand the underlying biology of patients with brain metastases or leptomeningeal metastases.^97,115^

The ability of EVs to drive tropism for particular metastatic sites in clinical samples was investigated by several groups to start to translate the previous preclinical data.^107,108^ Grigoryeva et al.^116^ investigated whether the presence of integrins β3, β4, and αVβ5 on EVS, CTCs and tumor cells associated with the site of development of metastatic disease in a cohort of breast cancer patients. With a cohort of 18 metastatic breast cancer patients and 48 nonmetastatic breast cancer patients (only 12 of whom developed metastatic disease during follow-up) they were able to confirm an association between EV-associated integrin β4 with the development of lung metastases but were unable to confirm an association between EV-associated integrin β3 and brain metastases due to the limited number of brain metastases patients in their cohort. Whereas, Chen et al.^117^ demonstrated a clear association between levels of EV-associated integrin β3 and worse overall survival as well as increased intracranial failure following whole brain radiotherapy in a cohort of 75 mixed subtype lung cancer patients. This association was unrelated to other clinical biomarkers known to be associated with worse outcomes including graded prognostic assessment scores.

In a much smaller study of 6 lung cancer patients (3 with brain metastases and 3 without), Wei et al.^118^ performed RNA sequencing of separated EVs and identified 22 differentially expressed miRNA including significant upregulation of miR-550a-3-5p in the plasma EVs of brain metastases patients which they subsequently validated using real-time quantitative PCR (RT–qPCR). Separately, Li et al.^119^ compared the proteomic cargo of plasma-derived EVs from lung cancer patients with solitary brain metastases (*n* = 26) solitary liver metastases (*n* = 16) and locally advanced lung cancer (*n* = 25) with healthy controls (*n* = 5). They identified 120 differentially expressed proteins in the EVS of brain metastase patients, of which MUC5B and SELL could be used as diagnostic biomarkers (AUC 0.774 and 0.720, respectively). Additionally, Carretero-González et al.^120^ prospectively collected plasma from a total of 123 patients with metastatic cancer (including 42 patients with brain metastases from lung cancer, breast cancer, kidney cancer, and melanoma and 31 healthy controls). Following EV separation, they characterized their EV sample including mass-spectrometry-based proteomics. In this study, they demonstrated a reduced concentration of EVs but a greater global protein concentration in patients with brain metastases and found an association between greater protein concentration with worse overall survival. They also investigated STAT3 and PDL1 levels by primary tumor subtype and demonstrated an association between greater EV-associated STAT3 levels in breast cancer brain metastases patients, but lower EV-associated STAT3 levels in melanoma brain metastases patients alongside greater EV-associated PDL1 levels in melanoma brain metastases patients. Furthermore, while not focusing on brain metastases specifically, several publications have demonstrated the utility of circulating EVs as monitoring biomarkers or response prediction for immune checkpoint inhibitor treatment in patients with metastatic NSCLC^121^ or metastatic melanoma^122^ which likely included patients with brain metastases.

Despite their potential, and some promising early results,^113^ blood-based EV biomarkers have yet to be translated into clinical practice. Despite several guidelines to improve the reliability of EV-related research and describing the necessary characterization steps,^112,123^ one significant challenge to clinical translation is the lack of standardization in the methods used to separate EVs, with several different techniques in frequent use in the scientific literature.^124^ However, this is improving, and our own work has identified that SEC-based separation seems to be the optimal EV separation technique from 1 mL plasma (*paper submitted*). This separation technique is relatively simple and can be performed using commercially available reagents facilitating its widespread adoption. The development of an optimized and standardized EV separation protocol from a 1mL plasma sample will facilitate a robust and reproducible analysis of EVs and their cargo, allowing the results to be more easily translated into clinical practice.

## Tumor-Educated Platelets

Additionally, the interrogation of TEPs is providing a novel source of liquid biopsy biomarkers.^30^ The link between platelets and malignancy is a well-known association, with thrombocytosis frequently identified in patients with advanced malignancies^125^ and high platelet counts linked with an increased risk of developing cancer.^126^ More recently, it has been identified that tumor cells can transfer biomolecules to platelets, thereby “educating” the platelets^127^ and highlighting that the assessment of platelet cargo could be used as a circulating biomarker.

Starting with Best et al.^128^ several publications from the same group have investigated the RNA transcript cargo of TEPs using a novel machine learning algorithm called thromboSeq as a liquid biopsy in a wide variety of cancers including a moderate cohort of patients with brain metastases. In a cohort of 126 brain metastase patients, primarily non-small cell lung cancer (*n* = 85), they demonstrated that TEP could differentiate between brain metastases and glioblastoma (a primary brain tumor) with an accuracy of 83–92%.^129^

While a subsequent study, including 93 patients with brain metastases, demonstrated that the TEP cargo of patients with brain metastases was distinct to patients with a similar primary tumor but without brain metastases, and that this cargo had similarities to primary brain tumor patients.^130^ They concluded that the TEP RNA cargo profiles seem to be influenced by the primary tumor and the metastatic site.

## Unanswered Questions

Despite their success in a variety of different settings in oncology,^100,131^ the more frequently investigated liquid biopsy techniques, including CTCs and cf/ctDNA have not been as successful when investigating brain tumors including brain metastases. Due to several biological factors, including the presence of the blood–brain barrier, the identification of brain metastases derived-CTCs or cf/ctDNA is reduced, obscured or absent compared to CTC or cf/ctDNA released by the primary tumor or metastases in other organs.^34,85^ Therefore, this suggests that a novel liquid biopsy analyte will be required to successfully develop a brain metastases-specific liquid biopsy biomarker.^72,132^

Additionally, there is a lack of studies comparing different liquid biopsy techniques for brain metastases. Given the different biological processes that result in the presence of the various liquid biopsy analytes in circulation, it is likely that the different biomarker information identified from each analyte may provide distinct biological information. Therefore, a combination of biomarkers might prove more effective than any individual analyte.

Finally, the biological processed underlying brain metastases development and progression are even more varied and distinct than their primary tumor of origin. In addition to common changes that occur in all brain metastases, it is therefore important to identify differences between brain metastases from different primary tumors and biological subtypes. This is especially important given the challenge in differentiating types of brain tumors, such as glioblastoma, primary CNS lymphoma, and brain metastases based on current imaging techniques let alone identifying subtypes of brain metastases.

## Conclusion

With an increasing variety of treatment options available, alongside improved survival for patients with brain metastases, there is a clear need for a more accessible and repeatable biomarker that can also provide a window into the underlying BM tumor biology as opposed to extracranial tumor biology. Advances in the technological assessment of liquid biopsy techniques over recent years are facilitating more reproducible investigations into their function from clinically relevant samples. However, with these outstanding questions, there has therefore never been a better time to study the role of extracellular vesicles and other liquid biopsy analytes, alone and in combination, in the biology of brain metastases and as strong candidates for biomarkers in brain metastases.

## Data Availability

All data generated or analyzed during this study are included in this published article.
